# Nutrient Acquisition and the Metabolic Potential of Photoferrotrophic Chlorobi

**DOI:** 10.3389/fmicb.2017.01212

**Published:** 2017-07-06

**Authors:** Katharine J. Thompson, Rachel L. Simister, Aria S. Hahn, Steven J. Hallam, Sean A. Crowe

**Affiliations:** ^1^Department of Microbiology and Immunology, University of British Columbia, VancouverBC, Canada; ^2^Departments of Earth, Ocean and Atmospheric Sciences, University of British Columbia, VancouverBC, Canada

**Keywords:** Chlorobi, Archean ocean, nitrogen, photoferrotrophy, sulfur

## Abstract

Anoxygenic photosynthesis evolved prior to oxygenic photosynthesis and harnessed energy from sunlight to support biomass production on the early Earth. Models that consider the availability of electron donors predict that anoxygenic photosynthesis using Fe(II), known as photoferrotrophy, would have supported most global primary production before the proliferation of oxygenic phototrophs at approximately 2.3 billion years ago. These photoferrotrophs have also been implicated in the deposition of banded iron formations, the world’s largest sedimentary iron ore deposits that formed mostly in late Archean and early Proterozoic Eons. In this work we present new data and analyses that illuminate the metabolic capacity of photoferrotrophy in the phylum Chlorobi. Our laboratory growth experiments and biochemical analyses demonstrate that photoferrotrophic Chlorobi are capable of assimilatory sulfate reduction and nitrogen fixation under sulfate and nitrogen limiting conditions, respectively. Furthermore, the evolutionary histories of key enzymes in both sulfur (CysH and CysD) and nitrogen fixation (NifDKH) pathways are convoluted; protein phylogenies, however, suggest that early Chlorobi could have had the capacity to assimilate sulfur and fix nitrogen. We argue, then, that the capacity for photoferrotrophic Chlorobi to acquire these key nutrients enabled them to support primary production and underpin global biogeochemical cycles in the Precambrian.

## Introduction

Modern global primary production is supported through oxygenic photosynthesis, which converts sunlight and CO_2_ into biomass, fuelling the biosphere and driving fluxes of matter and energy at global scales (Field et al., 1998). Primary production is limited by the availability of nutrients that are essential for growth such as phosphorus, nitrogen, and sulfur (Howarth, 1988). Primary producers thus expend valuable energy to meet their nutrient quotas. In the modern oceans, for example, cyanobacteria can fix nitrogen in the photic zone to support their nitrogen requirements (Karl et al., 1997). This in turn provides a competitive advantage that frequently allows nitrogen-fixing cyanobacterial species like *Trichodesmium* to outcompete non-nitrogen fixing species and can lead to cyanobacterial blooms (Capone and Carpenter, 1982; Capone et al., 2005). In addition to their role as primary producers in the modern oceans, cyanobacteria play a key role in the acquisition and redistribution of nutrients (Carpenter and Romans, 1991), driving global biogeochemical cycles since their evolution and proliferation in the Precambrian Eons.

Oxygenic photosynthesis and cyanobacteria emerged early in the Archean Eon (Crowe et al., 2013; Planavsky et al., 2014), evolving from anoxygenic phototrophs (Xiong et al., 2000), which arose as early as 3.8 Ga (Czaja et al., 2013). Like oxygenic phototrophs, anoxygenic phototrophs fix carbon dioxide into biomass, but instead of water as the electron donor they use a diverse set of inorganic species [e.g., H_2_, H_2_S, and Fe(II)] to replace electrons transferred from the photosystem to CO_2_ (Blankenship et al., 2006). Most anoxygenic phototrophs that grow in illuminated anoxic waters today use reduced sulfur species as their electron donors. During much of Earth’s early history, however, reduced sulfur species were likely scarce and the chemistry of marine sediments suggests that the oceans were overwhelmingly iron-rich (ferruginous) for long stretches of both the Archean and Proterozoic Eons (Canfield et al., 2008; Planavsky et al., 2011; Poulton and Canfield, 2011). Under these ferruginous conditions, ferrous iron would have been the most abundant and available inorganic electron donor (Canfield et al., 2006). Models for primary production in these ferruginous oceans suggest that anoxygenic phototrophs using Fe(II) as their electron donor—photoferrotrophs—could have supported up to 10% of modern day primary production before the proliferation of cyanobacteria (Canfield et al., 2006; Jones et al., 2015). Together, the evolutionary history of the photosystem and current knowledge on the history of ocean redox states imply that photoferrotrophs could have played a key role in driving global fluxes of matter and energy throughout the Precambrian Eons.

Compelling, but indirect, evidence for photoferrotrophy during Archean and Paleoproterozoic times comes from the deposition of banded iron formations (BIFs) (Garrels et al., 1973; Konhauser et al., 2002; Kappler et al., 2005). BIFs are massive iron ore deposits that were mostly deposited toward the end of the Neoarchean, though their deposition spans from the Eoarchean through to the Neoproterozoic Eras (Klein, 2005). Classical models for the deposition of iron from seawater to form BIF invoke large-scale oxidation of seawater Fe(II) by oxygen produced as a by-product of cyanobacterial growth and the subsequent precipitation and sedimentation of ferric iron minerals (Cloud, 1973; Garrels et al., 1973; Walker, 1987). Oxygen levels through the Archean, however, appear too low to support oxidation of Fe(II) at rates sufficient to sustain the rapid ferric Fe deposition needed to form even some of the apparently small BIFs like the Isua Greenstone belt in Greenland (Czaja et al., 2013). Instead, Fe(III) could have come from abiotic photochemical iron oxidation through UV photolysis (Garrels et al., 1973; Hartman, 1984), but this also appears too slow to support ferric iron deposition at rates recorded in BIFs (Konhauser et al., 2007). Alternatively, direct photosynthetic iron oxidation through photoferrotrophy could supply ferric Fe to form BIFs (Widdel et al., 1993; Konhauser et al., 2002). Accepting that oxygen levels were too low to drive Fe(II) oxidation and that UV photolysis appears similarly ineffective, photoferrotrophy may be the only viable mechanism to support appreciable ferric iron deposition and BIF formation. Nevertheless, the role of photoferrotrophs in BIF deposition remains controversial since direct evidence, like lipid biomarkers in BIFs, to diagnose photoferrotrophy, remain elusive. Extant cultures of photoferrotrophic bacteria are thus employed in efforts to further test the possible role of photoferrotrophs in BIF deposition and to identify signals that might be used to diagnose photoferrotrophy in the rock record.

A total of eight enrichments and isolates of photoferrotrophic bacteria have been brought into laboratory collections over the last 30 years. These cultures were largely obtained from a variety of benthic environments, such as marine mud flats and freshwater sediments (Widdel et al., 1993; Ehrenreich and Widdel, 1994; Heising and Schink, 1998; Heising et al., 1999; Straub et al., 1999), with a single isolate originating from a ferruginous water column (Llirós et al., 2015). Laboratory cultures of photoferrotrophs are distributed across the Alphaproteobacteria, the Gammaproteobacteria, and the Chlorobi and experiments conducted with these cultures reveal diverse physiological traits that translate to differential growth rates across a wide range of culture conditions (Kappler and Newman, 2004; Hegler et al., 2008; Posth et al., 2010). Notably, under modest light availability, many of these cultures grow sufficiently fast to oxidize Fe(II) at rates that would support the deposition of some of the largest BIFs (Konhauser et al., 2002; Kappler et al., 2005). This gives confidence in the capacity of photoferrotrophs to deposit BIFs, but laboratory culturing media are notoriously nutrient rich. Natural settings, on the other hand, are typically nutrient poor in comparison (Moore et al., 2013), and thus the role of photoferrotrophs in both BIF deposition and primary production would have depended on their capacity to grow and acquire nutrients from Precambrian seawater at concentrations almost certainly much lower than typical culture media.

Many laboratory experiments have been conducted with photoferrotrophs from the Alphaproteobacteria and Gammaproteobacteria (Kappler and Newman, 2004; Hegler et al., 2008; Posth et al., 2010; Bose and Newman, 2011; Pereira et al., 2012), but the ecological relevance of these groups in natural ferruginous settings is uncertain. In all modern ferruginous environments supporting photoferrotrophy, members of the Chlorobi appear to dominate (Crowe et al., 2008; Walter et al., 2014; Llirós et al., 2015). Furthermore, most or many extant photosynthetic communities dominated by anoxygenic phototrophs are comprised mostly of Chlorobi (Garrity et al., 2001). While anoxygenic photosynthesis by the Proteobacteria likely evolved early (Xiong et al., 2000), more recent phylogenomic analyses imply that the original phototrophs belonged to the Chlorobi (Sadekar et al., 2006; Bryant et al., 2012; Satoh et al., 2013). The reason for the apparent prevalence of the Chlorobi in modern environments is uncertain, but it is likely related to their ability to grow under environmentally relevant conditions including low nutrient availability and low light (Borrego and Garciagil, 1995; Manske et al., 2005; Gregersen et al., 2009; Romero-Viana et al., 2010; Biderre-Petit et al., 2011; Crowe et al., 2014a). Thus, despite the fact that photoferrotrophy by Proteobacteria may be relevant to Precambrian ecosystems, here, we focus our analyses on the Chlorobi because of their apparent ecological prominence in many modern systems and their deeper ancestry compared to phototrophic Proteobacteria.

Both phosphorus and nitrogen often limit photosynthetic activities and primary production in the modern oceans and in freshwater environments (Howarth, 1988). Phosphorus is generally considered the ultimate limiting nutrient on geological time scales as nitrogen can be fixed from the atmosphere when phosphorus is available (Tyrrell, 1999). Phosphorus is essential for life and is required in phospholipid, nucleic acid, and adenosine tri-phosphate (ATP) biosynthesis. Phosphorus throughout the Precambrian Eons was scarce with seawater concentrations orders of magnitude lower than today (Jones et al., 2015). This phosphorus scarcity would have led to low primary production, influencing the ecology and elemental stoichiometry of the photosynthetic primary producers (Reinhard et al., 2016). While phosphorus scarcity likely played an outsized role in shaping the Precambrian biosphere, nitrogen scarcity may have developed locally and transiently throughout the Precambrian Eons (Canfield et al., 2010; Michiels et al., 2017). Nitrogen is required to build essential cellular components such as DNA and amino acids. Biologically available nitrogen is supplied to the oceans through rock weathering and volcanism, but ammonium uptake and ultimate burial, however, would have eventually depleted the oceanic bioavailable nitrogen reservoir (Johnson and Goldblatt, 2015). In the modern ocean, biological fixation of atmospheric nitrogen keeps pace with phosphate supplies over geologic time scales (Moore et al., 2009). Nitrogen fixation is one of the most energetically expensive processes in the metabolic repertoire of life and yet it is distributed across distantly related groups of microorganisms (Meyer et al., 1978; Raymond et al., 2004). This underscores the importance of nitrogen fixation to microbial growth and production, is consistent with the early evolution and radiation of nitrogen fixation (Boyd et al., 2015; Stüeken et al., 2015; Weiss et al., 2016), and exemplifies how the distribution of core metabolic machinery across diverse lineages and functional guilds ensures survival of essential biogeochemical functions over geologic time (Falkowski et al., 2008). While the genomic potential for nitrogen fixation exists within the Chlorobi (Bryant et al., 2012), the capacity of photoferrotrophic Chlorobi to conduct nitrogen fixation and thus support Precambrian marine nitrogen quotas remains untested and unsubstantiated. This leaves our knowledge of the possible ecological role that photoferrotrophs may have played in the acquisition and redistribution of nitrogen and its attendant biogeochemical cycling in the Precambrian oceans entirely unknown.

In addition to phosphorus and nitrogen, sulfur is also essential for life and can limit biological production and growth when scarce (Da Silva and Williams, 2001). Sulfur on the modern Earth is abundantly available as the fully oxidized sulfate ion due to high concentrations of oxygen in the atmosphere and oceans, which promotes oxidative sulfur weathering and the recycling of sulfur from anoxic marine sediments. During the Precambrian Eons, however, marine sulfate concentrations were much lower (Walker and Brimblecombe, 1985; Crowe et al., 2014b) likely due to limited oxidative weathering and recycling under low O_2_ atmospheres (Walker and Brimblecombe, 1985; Habicht et al., 2002; Crowe et al., 2014b; Paris et al., 2014; Zhelezinskaia et al., 2014). Instead, sulfur was likely scarce and biologically available as low concentrations of sulfate, very low concentrations of sulfide, and possibly organic sulfur (Crowe et al., 2014b). Assimilatory sulfate reduction (ASR), therefore, would have been a key nutrient acquisition pathway, supporting primary production under low sulfur conditions. The genomic potential for ASR has been detected within two members the Chlorobi [*Chlorobium ferrooxidans* and *Chlorobium luteolum* (Frigaard and Bryant, 2008)], yet the role of ASR in photoferrotrophic growth remains uncertain. Photosynthetic growth of *C. ferrooxidans* on ferrous iron and without reduced sulfur compounds implies that the genomic potential for ASR translates into physiological capacity to convert sulfate into biomass sulfur (Heising et al., 1999). Given the likely low sulfate and extremely low sulfide concentrations perceived for the Precambrian oceans, ASR may have been absolutely critical for photoferrotrophs to operate as primary producers and contribute to a reservoir of biologically available reduced sulfur compounds in the ocean. The evolutionary history of ASR in the photoferrotrophic Chlorobi has not been explored, nor has sulfate uptake been quantitatively assessed. The role of photoferrotrophs in driving sulfur cycling during the Precambrian remains underappreciated and untested creating another gap in our knowledge of nutrient acquisition and redistribution in the Precambrian oceans.

To address the response of photoferrotrophy to nitrogen and sulfur scarcity, and to create new knowledge relevant to nitrogen and sulfur acquisition and redistribution in the Precambrian oceans, we examined two extant demonstrably photoferrotrophic Chlorobi: benthic *C. ferrooxidans* (grown in co-culture with *Geospirillum* sp. KoFum) (Heising et al., 1999), and pelagic *Chlorobium phaeoferrooxidans* (Llirós et al., 2015; Crowe et al., 2017). We also examined putative benthic photoferrotroph *C. luteolum*, postulated to grow through photoferrotrophy because of its genomic potential for ASR (Frigaard and Bryant, 2008). We verified the capacity of photoferrotrophic Chlorobi to fix inorganic nitrogen and sulfur, and constrained the antiquity of this capacity in the Chlorobi through phylogenetic analyses.

## Results and Discussion

### Nitrogen Fixation

The process of fixing dinitrogen is kinetically challenging and energetically expensive as it involves overcoming the activation energy required in breaking the triple bond between the two nitrogen molecules. The enzyme necessary for nitrogen fixation, nitrogenase, is a multi-subunit protein that is assembled and regulated by a series of other related proteins. All nitrogenases require a metal ion cofactor – molybdenum, iron, or vanadium – with each cofactor being recruited and incorporated into the nitrogenase by a different set of proteins, Nif, Anf, and Vnf, respectively. Current studies indicate that the majority of nitrogenases depend on the molybdenum ion cofactor for their enzymatic activity (reviewed in Rubio and Ludden, 2008), while the iron and vanadium dependant nitrogenases may play a role in molybdenum limiting environments (Joerger et al., 1988). Phylogenetic evidence suggests that the molybdenum-dependant version of the enzyme evolved first (Boyd et al., 2011), which is further supported by the observation that organisms identified as having an iron or vanadium dependant nitrogenase all contain a copy of the molybdenum-dependant nitrogenase (Raymond et al., 2004; Soboh et al., 2010). There are up to 25 proteins, depending on the species, required to assemble and regulate the nitrogenase including three conserved structural proteins: NifD, NifK, and NifH. NifH is often used as the marker gene for nitrogen fixation in natural environments, due to its role in the main enzyme structure and in cofactor recruitment. Further phylogenetic information, however, can be obtained when all three structural proteins (NifDKH) are concatenated due to increased sequence information and the conserved nature of all three proteins. Here we explored these key structural proteins to test for the metabolic potential for nitrogen fixation in the photoferrotrophic Chlorobi. We compare nitrogen fixation in photoferrotrophic Chlorobi to the other members of the phylum Chlorobi and to representatives from all phyla capable of nitrogen fixation to assess the evolutionary history of nitrogenase in relevant to photoferrotrophy in the Chlorobi and to place constraints on the possible role of photoferrotrophs in supplying fixed nitrogen to the Precambrian oceans.

#### Distribution of Nitrogen Fixation Pathways within Chlorobi

**FIGURE 1:**
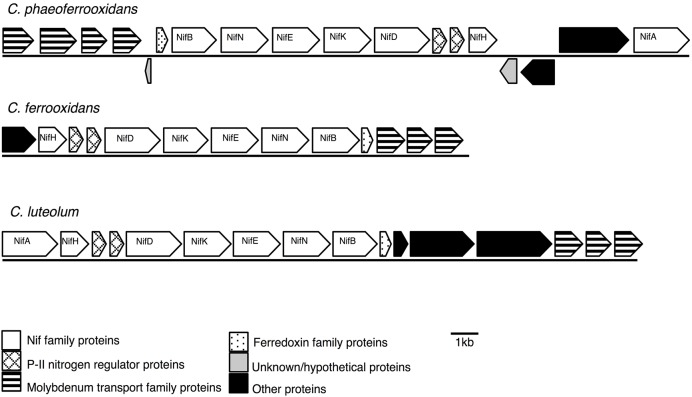
Nitrogenase gene cassettes of the photoferrotrophic Chlorobi, detailing the position of each gene and the differences and similarities between the gene cassettes.

Previous analyses of Chlorobi genomes identified that the metabolic capacity for nitrogen fixation is distributed across the phylum with the exception of the *Ignavibacterium* sp. (Bryant et al., 2012; Liu et al., 2012; Hiras et al., 2016). *Ignavibacterium* sp. is the deepest branching member of the Chlorobi and the only class of non-photosynthetic organisms in the phylum. Here we show that genes coding for the proteins required for nitrogen fixation are present in the genomes of the photoferrotrophic Chlorobi *C. ferrooxidans* and *C. phaeoferrooxidans*, putative photoferrotroph *C. luteolum* (**Figure 1**), and in genomes of all other members of the Chlorobi (data not shown). Specifically, we identified one homolog of each of the molybdenum-dependant nitrogenase proteins in all three photoferrotrophic Chlorobi. No homologs of the alternative vanadium or iron-only nitrogenase proteins were detected (PSI-Blast, expect threshold 10). These results indicate that the photoferrotrophic Chlorobi have the genomic capacity to fix nitrogen. Furthermore, nitrogen fixation is wide spread among the Chlorobi, with all available Chlorobi genome sequences coding the necessary proteins apart from *Ignavibacterium album*.

#### Biochemical Verification of Nitrogen Fixation

**FIGURE 2:**
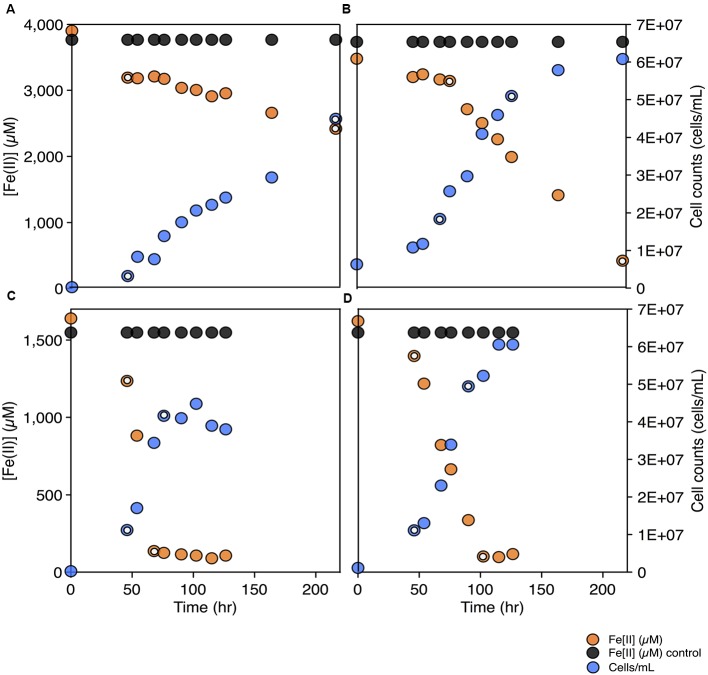
Fe(II) concentrations and cell counts over time for both *C. phaeoferrooxidans* **(A,C)** and *C. ferrooxidans* **(B,D)** under two sets of media: no bioavailable nitrogen – N_2_ as sole nitrogen source **(A,B)** and ammonium rich **(C,D)**. Data points used to calculate growth rates and Fe(II) oxidation rates are highlighted in each panel.

To test for the biochemical capacity to fix nitrogen during photosynthetic growth on Fe(II), nitrogen free (below limit of detection ammonium, ammonia, nitrate, or nitrite) media was inoculated with *C. phaeoferrooxidans* or *C. ferrooxidans*. Both species were also grown in the standard growth medium containing 5.6 mM ammonium (Hegler et al., 2008), for comparison. Both species were able to fix nitrogen while growing through photosynthetic Fe(II) oxidation with doubling times of 45 and 36 h for *C. phaeoferrooxidans* and *C. ferrooxidans*, respectively (**Figures 2A,B**). Fe(II) oxidation rates, during exponential growth phase, were 4.8 ± 0.33 μM/hour (*C. phaeoferrooxidans*) and 16 ± 0.56 μM/hour (*C. ferrooxidans*) (**Figures 2A,B**). Growth under ammonium-rich conditions supported shorter doubling times (15 and 27 h) and higher rates of Fe(II) oxidation (50 ± 2.4 μM/hour and 23 ± 0.7 μM/hour) for *C. phaeoferrooxidans* and *C. ferrooxidans*, respectively (**Figures 2C,D**). These results indicate that both pelagic *C. phaeoferrooxidans* and benthic *C. ferrooxidans* are capable of using dinitrogen gas as their sole source of nitrogen during growth, but that the need to fix N decreases growth rates.

To further explore the metabolic capacity of photoferrotrophic Chlorobi under both sets of conditions, cell specific Fe(II) oxidation rates were calculated for each species. *C. phaeoferrooxidans* oxidized Fe(II) at 21.2 ± 1.4 fmol/cell while fixing nitrogen and 47.8 ± 2.3 fmol/cell under ammonium-rich conditions. Conversely, *C. ferrooxidans* oxidized Fe(II) at 30.0 ± 0.9 fmol/cell and 29.4 ± 1.0 fmol/cell in ammonium free and ammonium-rich media, respectively, with no appreciable difference during N-fixation. The apparent insensitivity of *C. ferrooxidans* to N-availability may be related to the presence of its co-culture partner, *Geosprillum* sp. KoFum. Further experiments with KoFum could help constrain its possible role in N metabolism within the co-culture The observation that *C. phaeoferrooxidans* has lower cell specific growth rates under N scarcity, however, implies lower growth yields during N fixation. Both species are ultimately capable of growth and Fe(II) oxidation while fixing nitrogen but the differential response of cell specific iron oxidation rates to N-scarcity implies that nutrient availability can influence the ecology of photoferrotrophs in the environment.

#### Evolutionary History of Nitrogen Fixation in the Chlorobi

To assess the evolutionary history of nitrogen fixation in the Chlorobi we tested for horizontal gene transfer (HGT) within the photoferrotrophic Chlorobi and conducted phylogenetic analyses of Nif proteins, which we compared to small subunit 16S ribosomal RNA (SSU rRNA) genes. Deviations in the branching orders between these phylogenies would indicate non-vertical inheritance and HGT. To test for horizontal transfer of Nif genes in the photoferrotrophic Chlorobi, we looked for characteristic signatures of HGT within *nif* gene cassettes. Codon adaptation index (CAI) values, a metric used to describe differences in codon usage between specific genes and the genomic background, were calculated for all individual *nif* genes belonging to *C. ferrooxidans*, *C. phaeoferrooxidans*, and *C. luteolum*. All CAI values were greater than the threshold value, 0.70, below which HGT is indicated (**Table 1**). In addition, GC contents of *nif* genes were very similar to GC contents of genomic backgrounds providing no evidence for HGT (**Table 1**). Our analyses also failed to identify tRNAs, transposases, or other genetic elements commonly associated with gene mobility in close proximity (within 5000 bp) to the nitrogenase gene cassette in any of the photoferrotrophic Chlorobi. The general lack of tRNAs or transposases near the *nif* cassettes in the photoferrotrophic Chlorobi, combined with super threshold CAI values and *nif* gene GC contents that are homogenous against genomic backgrounds, imply *nif* gene acquisition through vertical decent.

**Table 1:** Gene length (bp), codon adaptation index (CAI), and GC content (%) for each of the genes in the nitrogenase cassette.

Gene	*C. phaeoferrooxidans*			*C. ferrooxidans*			*C. luteolum*		
	Length (bp)	CAI	GC content (%)	Length (bp)	CAI	GC content (%)	Length (bp)	CAI	GC content (%)
NifB	1275	0.80	52.63	1275	0.76	53.18	1263	0.75	60.89
NifN	1353	0.79	53.22	1350	0.75	53.48	1353	0.74	59.42
NifE	1362	0.79	50.07	1362	0.75	49.63	1362	0.74	57.34
NifK	1383	0.81	53.51	1383	0.73	52.78	1380	0.76	60.14
NifD	1635	0.79	49.54	1635	0.76	49.66	1641	0.77	57.22
PII regulator	378	0.81	50.00	378	0.72	50.00	378	0.72	59.26
PII regulator	357	0.76	49.30	357	0.73	48.74	357	0.70	56.30
NifH	825	0.83	49.58	825	0.79	49.21	825	0.81	59.03

*Each parameter was calculated for all three photoferrotrophic Chlorobi, whose whole genome GC contents are: 49.72% for Chlorobium phaeoferrooxidans, 49.9% for C. ferrooxidans, and 58.1% for C. luteolum*.

**FIGURE 3:**
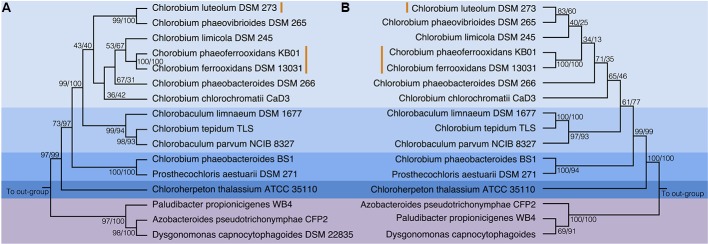
Phylogenies of the Chlorobi and Bacteroidetes using **(A)** the concatenated NifDKH proteins and **(B)** 16S rRNA with bootstrap values shown at each node (maximum likelihood/maximum parsimony). The blue colors delineate the organisms of the Phylum Chlorobi, with each shade representing a different genus, while the purple color delineates the Phylum Bacteroidetes. The orange lines indicate the position of the photoferrotrophic Chlorobi. The trees were rooted with four cyanobacterial organisms. Note: *Azobacteroides pseudotrichonymphae* CFP2 is abbreviated from Candidatus *Azobacteroides pseudotrichonymphae* genomovar. CFP2.

To test the evolutionary history of the *nif* genes in the Chlorobi, we conducted phylogenetic analyses of concatenated NifDKH proteins of all cultured and sequenced Chlorobi that have the genomic potential to fix dinitrogen. The Chlorobi sequences were aligned with selected sequences from the next closest phylum – Bacteroidetes – and the tree was rooted using four Cyanobacterial species as an out-group (**Figure 3A**). The genus *Chlorobium*, which includes all three photoferrotrophic Chlorobi, the genus *Chlorobaculum*, and the genus *Prosthecochloris* all form monophyletic groups that are collectively part of the phylum Chlorobi clade. Likewise, the Bacteroidetes form a monophyletic group and share a common ancestor with the members of the phylum Chlorobi. Furthermore, when the NifDKH tree is compared to a 16S rRNA tree of the same organisms (**Figure 3B**), all of the genera within the phylum Chlorobi branch in an identical order to those in the 16S rRNA phylogeny. The phylogenetic relationship between the Chlorobi and Bacteroidetes is the same for both NifDKH and 16S rRNA sequences, indicating that the common ancestor to the phyla Chlorobi and Bacteroidetes likely contained a nitrogenase and therefore the ability to fix dinitrogen. *Ignavibacterium* sp., the sole members of the phylum Chlorobi who do not posses a nitrogenase, likely lost the capability to fix nitrogen as the remainder of the Chlorobi and the phylum Bacteroidetes bracket the phylogenetic position of the *Ignavibacterium* sp. Taken together, available data imply vertical decent.

Accepting largely vertical descent of NifDKH from the common ancestor of the Chlorobi and Bacteroidetes, NifDKH must have emerged within this line of descent before the divergence of the Chlorobi and Bacteroidetes. The timing of this divergence has been estimated using a whole genome molecular clock (David and Alm, 2011) to between 3 and 1.6 Gya, which implies the capacity to fix N in the ancestors of the Chlorobi before this time. Independent N isotope data from metasedimentary kerogen implies N fixation by at least 3.2 Gy (Stüeken et al., 2015). Combined, the evidence for vertical inheritance of NifDKH in the Chlorobi on the taxonomic levels of genus and phylum, the timing of divergence between the Chlorobi and the Bacteroidetes, and the N isotope record, imply that ancestors of modern Chlorobi likely had capacity to fix nitrogen in the iron-rich oceans of the paleoproterozoic and perhaps as early as the mesoarchean eras.

**FIGURE 4:**
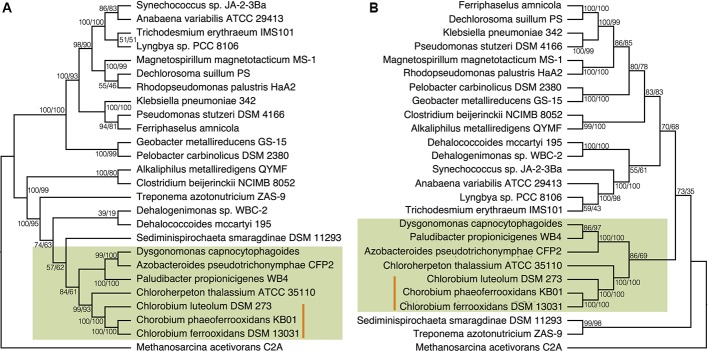
Phylogenies of **(A)** the concatenated NifDKH proteins and **(B)** 16S rRNA for two to four representatives of several nitrogen-fixing phyla with bootstrap values shown at each node (maximum likelihood/maximum parsimony). The green color delineates the Chlorobi/Bacteroidetes monophyletic grouping, while the orange line indicates the position of the photoferrotrophic Chlorobi. Note: *Azobacteroides pseudotrichonymphae* CFP2 is abbreviated from Candidatus *Azobacteroides pseudotrichonymphae* genomovar. CFP2.

To place N fixation in the Chlorobi, and Bacteroidetes, within the broader context of nitrogenase evolution in general, we conducted further phylogenetic analyses using a greater diversity of organisms. We analyzed the NifDKH phylogeny using two to four representatives from every phylum that had a cultured and sequenced species with previously documented genomic potential for nitrogen fixation (**Figure 4A**). This phylogeny places the Nif proteins found in the Chlorobi and Bacteroidetes in a single clade, supporting their emergence from a common ancestor and the vertical inheritance of NifDKH from this ancestor. The phylogeny of the NifDKH protein is, however, incongruent with that of the 16S rRNA gene from the same organisms (**Figure 4B**). While the Chlorobi and Bacteroidetes group together in both phylogenies, the Spirochetes, Chloroflexi, and Firmicutes also group with the Chlorobi in the NifDKH phylogeny, but belong to distinct clades in the 16S rRNA gene phylogeny. The differences between these phylogenies confound further constraints on the evolutionary history of nitrogenase within the Chlorobi based on phylogeny and add to the overwhelming evidence for horizontal transfer of NifDKH genes (Raymond et al., 2004; Falkowski et al., 2008; Boyd and Peters, 2013).

#### Ecology of Nitrogen Fixation in Chlorobi, Past and Present

Members of the phylum Chlorobi underpin biological production in many modern anoxic environments, both sulfidic (Overmann, 1997; Tonolla et al., 2004; Gregersen et al., 2009; Kondo et al., 2009; Meyer et al., 2011) and ferruginous (Walter et al., 2014; Llirós et al., 2015), through their ability to harness light energy and fix inorganic carbon into biomass, even at low light intensities (Manske et al., 2005). Chlorobi further contribute to biogeochemical cycling in these systems through the acquisition and redistribution of essential nutrients, such as nitrogen. This ecological role would have extended to global scales in the low oxygen Precambrian oceans. Our analyses confirm the genomic potential to fix N in all but one of the Chlorobi lineages and directly demonstrate the capacity of the photoferrotrophic Chlorobi to fix dinitrogen as their sole source of nitrogen while oxidizing Fe(II). Rates of Fe(II) oxidation are, however, slower when photosynthetic growth is supported through N-fixation rather than ammonium assimilation. To test the impact of slower rates of Fe(II) oxidation, and therefore growth, on the deposition of BIFs, we ran our cell counts and Fe(II) oxidation rates through the calculation outlined by Konhauser et al. (2002). Our data indicates that both photoferrotrophic strains would be capable of generating even the largest BIFs (i.e., the Hamersley BIF) with maximum of 2.44 × 10^3^ photoferrotrophic cells/mL required in the basin. Thus, photoferrotrophic growth coupled to N-fixation could support BIF deposition, even in the face of nitrogen scarcity.

*Chlorobium phaeoferrooxidans*, and *C. ferrooxidans* exhibit differential responses to N scarcity that manifest in different cell specific Fe(II) oxidation rates and different ratio’s between microbial growth (cell doubling times) and Fe(II) oxidation. *C. phaeoferrooxidans* has a cell doubling time to Fe(II) oxidation ratio of 9.4 under N-fixing conditions compared to 0.3 when there is ample ammonium, whereas *C. ferrooxidans* has comparable ratio’s of 2.3 and 1.2 for N-fixing and ammonium-rich conditions comparatively. This differential response indicates that under ammonium-rich conditions *C. phaeoferrooxidans* grows more efficiently (i.e., with a higher growth yield) whereas when N-fixation is required *C. ferrooxidans* grows more efficiently. This creates niches for each microorganism defined by N availability. The differential response also implies that the stoichiometry of Fe-oxidation to biomass production and cell growth is partly decoupled and depends on N availability. Essentially, this decoupling means that more Fe(II) is oxidized to produce an individual cell during growth supported by N-fixation than by ammonium assimilation. Such a decoupling thus requires either the diversion of reducing equivalents (NADH) produced during photosynthesis into compounds not used directly in cell growth, or that cell growth and division requires more fixed carbon during N-fixation. The former could include conversion of N_2_ to ammines and the biosynthesis of cell exudates, and the latter might include the biosynthesis of cellular proteins needed to conduct N-fixation. Such a decoupling would influence the overall biogeochemical functioning and ecology of ecosystems supported through primary production by photoferrotrophy. The overall activity of the marine biosphere through the Precambrian Eons may thus have been influenced by the availability of fixed N to photoferrotrophs.

### Assimilatory Sulfate Reduction (ASR)

Sulfate ions are biologically inert and organisms expend tremendous energy ‘activating’ sulfate for three main functions: (1) reduction and incorporation into amino acids; (2) condensation and incorporation into sulfolipids and other small molecules; and (3) for dissimilatory sulfate respiration. In addition to the reduction of sulfate, organisms can acquire organic sulfur compounds like amino acids, and hydrogen sulfide directly from the environment. Acquisition of these reduced sulfur compounds can considerably reduce the expenditure of energy on sulfur acquisition. Here we focus on the first two assimilatory pathways and the capacity for reductive sulfur assimilation in the photoferrotrophic Chlorobi. The proteins required to complete an entire ASR pathway include: CysD, the sulfateadenyl transferase that activates sulfate to form APS; CysN which catalyzes GTP hydrolysis providing the energy needed to adenylate imported sulfate; CysC (a domain of CysN), the APS kinase that phosphorylates APS to PAPS; and CysH, the APS reductase which reduces the sulfur in APS to sulfite. We have explored the metabolic potential for sulfate assimilation in the genomes of the photoferrotrophic Chlorobi and directly tested sulfate incorporation into biomass.

#### Distribution of ASR Pathways within Chlorobi

**FIGURE 5:**
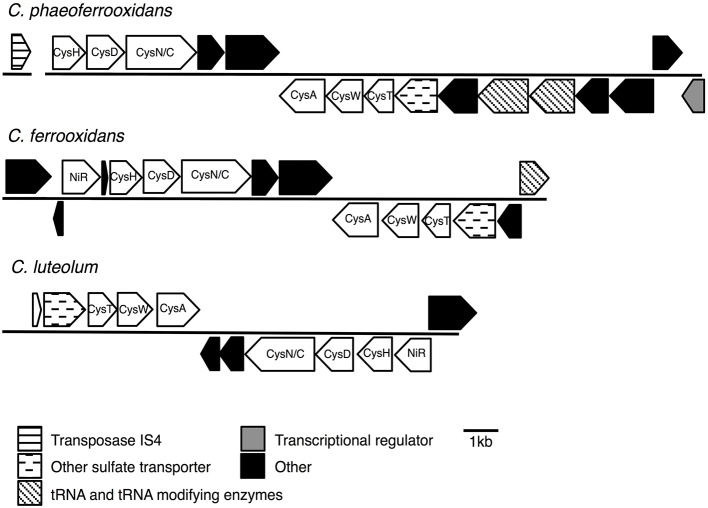
Assimilatory sulfate reduction (ASR) gene cassettes for the photoferrotrophic Chlorobi, detailing the position of each gene and the differences and similarities between the gene cassettes.

Previous analyses of Chlorobi genomes identified the metabolic capacity for ASR in *C. ferrooxidans* and *C. luteolum* (Frigaard and Bryant, 2008). Using all currently available genomic information we identified components of the ASR pathways distributed throughout the Chlorobi (**Table 2**). We find that the photoferrotrophic Chlorobi, *C. ferrooxidans* and *C. phaeoferrooxidans*, as well as putative photoferrotroph *C. luteolum*, all possess the necessary proteins for ASR – CysD, CysN/C, CysH – and therefore have the potential capacity to synthesize amino acids from exogenous sulfate (**Figure 5**). Notably, the presence of both CysD and CysN indicate that sulfate activation to APS in these Chlorobi is coupled to GTP hydrolysis. Sulfate assimilation in the Chlorobi, therefore, offsets the energetic expense associated with sulfate activation. The presence of CysN/C indicates the metabolic potential to phosphorylate APS to PAPS implying that these strains might have capacity to synthesize sulfate-containing compounds like sulfolipids. Finally, while components of assimilatory sulfate metabolisms are more broadly distributed throughout the Chlorobi, genes coding for key components of the pathway are mostly missing implying a lack of capacity for sulfate assimilation outside the photoferrotrophic Chlorobi (**Table 2**). Given the metabolic potential for ASR in the photoferrotrophic Chlorobi, we sought to biochemically verify this process.

**Table 2:** Assimilatory (orange) and dissimilatory (blue) sulfur proteins present in the genomes of green sulfur bacteria.

Organism	CysD	Sat	CysNC	CysH	AprAb	DsrAB
*Chlorobium phaeoferrooxidans* KB01	+	-	+	+	-	-
*Chlorobium ferrooxidans* DSM 13101	+	-	+	+	-	-
*Chlorobium luteolum* DSM 273	+	-	+	+	-	+
*Chlorobium phaeovibrioides* DSM 265	+	-	+	-	-	+
*Prosthecochloris aestuarii* DSM 271	+	-	+	-	-	+
*Chlorobium phaeobacteroides* BS1	-	+	+	-	+	+
*Chlorobium chlorochromatii* CaD3	-	+	+	-	+	+
*Pelodictyon phaeoclathratiforme*	-	+	-	-	+	+
*Chlorobium tepidum* TLS	-	+	-	-	+	+
*Chlorobium phaeobacteroides* DSM 266	-	-	-	-	-	+
*Chlorobium limicola* DSM 245	-	-	-	-	-	+
*Chlorobaculum parvum* NCIB 8327	-	-	-	-	-	+
*Chloroherpeton thalassium*	-	-	-	-	-	-
*Ignavibacterium album*	-	-	-	-	-	-

#### Biochemical Verification of ASR

*Chlorobium phaeoferrooxidans* and *C. ferrooxidans* are both known to grow in media where sulfur is supplied exclusively in the form of sulfate, which directly demonstrates the physiological capacity for sulfate assimilation. We quantitatively tested this capacity by measuring the uptake of ^35^S labeled sulfate in low sulfate growth media. *C. phaeoferrooxidans*, indeed took up ^35^S labeled sulfate into TCA extractable biomass, demonstrating assimilatory reduction of sulfate and its incorporation into amino acids. Over the course of these sulfate uptake experiments, *C. phaeoferrooxidans* oxidized 3070 μM Fe(II). This implies the fixation of 770 μM C, based on the 4:1 stoichiometry between Fe(II) oxidation and C fixation observed for *C. phaeoferrooxidans* during growth on Fe(II), and for photoferrotrophic organisms, more generally (Widdel et al., 1993). A corresponding total of 3 μM S was fixed demonstrating a ratio of 260:1 C to S, which we take as approximately indicative of the S content of *C. phaeoferrooxidans*. There are few data to compare with, but our results suggest that *C. phaeoferrooxidans* has relatively low S quotas compared to aquatic and cultured bacteria (C:S from 10–60) (Fagerbakke et al., 1996) and particulate organic matter from the North Pacific (C:S of 50) (Chen et al., 1996). By analogy to *C. phaeoferrooxidans*, photoferrotrophic Chlorobi likely have capacity to fix sulfate into biomass under low sulfate conditions, which they appear well adapted to do based on minimal cellular sulfur quotas in comparison to other bacteria and marine organic material.

#### Evolutionary History of ASR in the Chlorobi

To test for horizontal transfer of ASR genes to the photoferrotrophic Chlorobi, we searched for characteristic signatures of HGT within the ASR cassettes and conducted phylogenetic analyses of ASR genes, which we compared to 16S rRNA gene phylogenies. The CAI value for each of the ASR genes belonging to *C. ferrooxidans* and *C. phaeoferrooxidans* were all greater than the threshold value, 0.70, below which HGT is indicated (**Table 3**). ASR genes in *C. luteolum*, however, had sub-threshold CAI values, as low as 0.53, indicating possible ASR gene acquisition through horizontal transfer. The GC contents of ASR genes for all three species were very similar to GC contents of their respective genomic backgrounds, providing no evidence for HGT (**Table 3**). Collectively, these data provide little evidence for the lateral acquisition of ASR gene cassettes in the photoferrotrophic Chlorobi, although the evidence for vertical descent is greater in *C. phaeoferrooxidans* and *C. ferrooxidans* than in *C. luteolum*. A single transposase (**Figure 5**) was found on a contig adjacent to that hosting the ASR gene cassette in *C. phaeoferrooxidans*. The general lack of tRNAs or transposases near the ASR cassettes in the photoferrotrophic Chlorobi combined with super threshold CAI values and ASR gene GC contents that are homogenous against the genomic backgrounds, implies ASR gene acquisition through vertical decent.

**Table 3:** Gene length (bp), CAI, and GC content (%) for each of the genes in the ASR cassette.

Gene	*C. phaeoferrooxidans*			*C. ferrooxidans*			*C. luteolum*		
	Length (bp)	CAI	GC content (%)	Length (bp)	CAI	GC content (%)	Length (bp)	CAI	GC content (%)
CysH	714	0.78	54.34	714	0.78	54.62	753	0.59	56.97
CsyD	882	0.80	56.12	882	0.78	55.56	915	0.61	57.38
CsyN/C	1800	0.81	54.28	1800	0.77	53.67	1800	0.69	58.06
Siroheme synthase	453	0.81	54.08	453	0.71	52.98	453	0.53	57.17
Uroporphyrin-III C-methyltransferase	1287	0.73	57.96	1287	0.73	55.40	258	0.62	57.36
CysA	1074	0.77	53.26	1074	0.80	52.42	1074	0.67	59.22
CysW	870	0.79	53.22	870	0.79	52.76	870	0.64	58.74
CysT	834	0.79	52.64	834	0.81	53.36	834	0.66	58.03
Sulfate transporter	1020	0.82	53.14	1020	0.79	52.65	1008	0.75	59.52

*Each parameter was calculated for all three photoferrotrophic Chlorobi, whose whole genome GC contents are: 49.72% for C. phaeoferrooxidans, 49.9% for C. ferrooxidans, and 58.1% for C. luteolum.*

**FIGURE 6:**
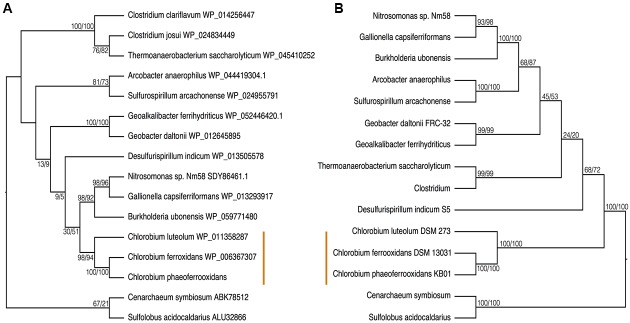
Phylogenies of **(A)** the CysH protein and **(B)** 16S rRNA with bootstrap values shown at each node (maximum likelihood/maximum parsimony). The orange line indicates the position of the photoferrotrophic Chlorobi. The trees are rooted with two Archaeal species.

To further test the evolutionary history of ASR, the CysH protein was analyzed to examine the phylogenetic relationship between the proteins used in the photoferrotrophic Chlorobi and ASR in other organisms. The photoferrotrophic Chlorobi grouped together forming a monophyletic clade within the CysH phylogeny (**Figure 6A**). The photoferrotrophic Chlorobi exhibit congruent phylogenies between the CysH protein and 16S rRNA gene (**Figure 6B**), providing further evidence in support of vertical inheritance of the ASR pathway in the photoferrotrophic Chlorobi. The more general evolutionary history of the CysH protein, however, is convoluted given abundant incongruences between the CysH protein and 16S rRNA gene phylogenies. Accepting vertical inheritance of the ASR pathway in the photoferrotrophic Chlorobi and the early divergence of the Chlorobi from other organisms, we hypothesize that gene loss explains the lack of a complete ASR pathways in other photosynthetic Chlorobi and this hypothesis is supported by the partial presence of ASR pathway components across the phylum Chlorobi (**Table 2**).

#### Ecology of ASR in Chlorobi, Past and Present

The presence of an ASR pathway in all known photoferrotrophic Chlorobi implies that ASR is advantageous to growth under ferruginous conditions. The lack of the ASR pathway in the canonically sulfur oxidizing Chlorobi makes sense in light of the availability of reduced sulfur compounds in their preferred habitats. The energetic expense of ASR would tend to favor assimilation of reduced compounds when available. Conversely, ferruginous environments are by definition sulfur poor and the availability of reduced sulfur compounds can be limited by the solubility of FeS. Sulfate, therefore, is likely the most abundant and available sulfur source in modern ferruginous environments. The rock record also demonstrates that ferruginous marine conditions persisted throughout much of the Precambrian Eons and reduced sulfur species were likely scarce with the exception of in the apparently ephemeral developments of costal euxinia. ASR may thus have supported sulfur requirements of photoferrotrophic primary producers over long stretches of Earth’s history.

The apparent role of ASR in supporting primary production through photoferrotrophy implies that sulfate availability could have been an important control on global productivity. At 28 mM, sulfate is the principle anion in modern seawater, but sulfate concentrations could have been as low as a few μM in the Archean oceans (Crowe et al., 2014b). Nutrients like phosphorus and nitrogen are known to become limiting at such low concentrations. The apparently low sulfur quotas of the photoferrotrophic Chlorobi (260:1, C:S) thus seem well adapted to growth in the low sulfate oceans of the Archean, which would have enhanced productivity in the face of sulfur scarcity.

Under low sulfate conditions dissimilatory sulfate reduction (DSR) would have played a comparatively small role in the remineralization of organic matter in Archean oceans (Crowe et al., 2014b). Qualitatively then ASR would have played an outsized role in the reduction of sulfur and the global sulfur cycle in the Archean oceans, relative to today. We therefore hypothesize that primary production through photoferrotrophy was a key pathway in the production of an organic reduced sulfur pool, which would have provided an important vector for sulfur to Archean sediments. We further hypothesize that ASR may have predated DSR. Earliest evidence for DSR comes from S-isotope fractionation recorded in 3.47Gya barites (Shen et al., 2001), whereas photoferrotrophy likely operated as early as 3.8Gya (Czaja et al., 2013) and presumably required ASR. The idea that ASR predates DSR could be tested if homology could be established in enzymes involved in both pathways. Although ASR and DSR serve different functions – sulfate acquisition versus energy transduction, respectively – both pathways actively transport sulfate into the cell and the first enzymes in the pathways are thus analogous. Comparison of amino acid sequences of the first enzymes (CysD and Sat, respectively) in the two pathways indicates a strong degree of homology implying evolutionary relationships between components of ASR and DSR.

**FIGURE 7:**
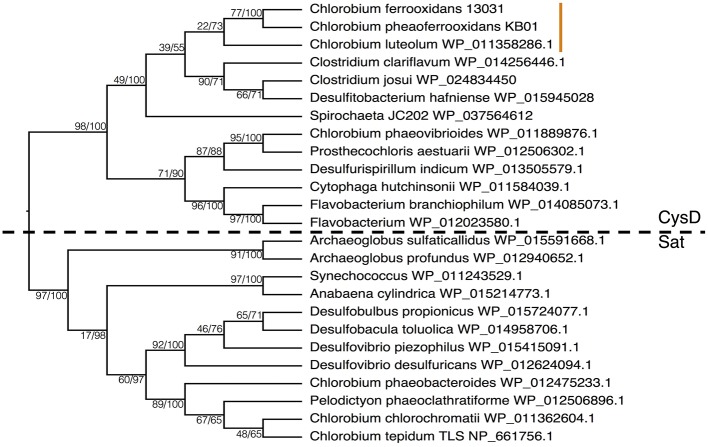
Phylogeny of the Sat/CysD protein with bootstrap values shown at each node (maximum likelihood/maximum parsimony), to compare the ASR and DSR pathway among a diverse set of organisms. The dashed line delineates the organisms with CysD versus those with Sat. The orange line indicates the position of the photoferrotrophic Chlorobi.

To examine the phylogenetic relationships between enzymes that transport sulfate for use in ASR and DSR, we aligned CysD and Sat proteins from the majority of the Chlorobi and a selection of representative microorganisms from diverse phyla. The resulting phylogeny clearly separated amino acid sequences annotated as CysD from those annotated as Sat (**Figure 7**). Both CysD and Sat appear to support sulfate transport in relation to multiple sulfur metabolisms, but the phylogenetic relationships appear complicated and likely require more detailed analyses. Nevertheless, homology between the two proteins implies a possible evolutionary relationship between ASR and DSR that may inform evolutionary histories and should be tested in the future.

## Outlook

Photoferrotrophy links the C and Fe biogeochemical cycles through coupled CO_2_ fixation and Fe(II) oxidation and has likely done so since the early Archean Eon. Models for photoferrotrophic growth in the Archean oceans remain poorly constrained as they are extrapolated from growth rates in nutrient rich laboratory culture media. Here we demonstrate that photoferrotrophic Chlorobi have the physiological capacity to fix inorganic N and S into biomass when availability of these nutrients is low and have likely had this capacity since the Archean Eon. Thus, under N and S limited ferruginous conditions, photoferrotrophy underpins biogeochemical cycling of C, N, S, and Fe. Nutrient availability, however, influences growth and Fe(II) oxidation rates and has consequences for the stoichiometric relationships between C, N, S, and Fe transformations. Undoubtedly, these relationships should be assessed and studied in more detail with additional physiological experimentation and should be applied to further constrain models of photoferrotrophy, biological production, and global biogeochemical cycling in the Archean Eon.

## Materials and Methods

### Strains and Growth Medium

Media was prepared after Hegler et al. (2008), and allocated into serum bottles (100 mL media and 160 mL total volume), with 0.3 g/L NH_4_Cl, 0.5g/L MgSO_4_⋅7H_2_O, 0.1g/L CaCl_2_⋅2H_2_O, and 0.6g/L KH_2_PO_4_. After autoclaving, 22 mmol L^-1^ bicarbonate, trace elements, mixed vitamin solution, selenate-tungstate, vitamin B12, and FeCl_2_ were added and the pH was adjusted to 6.8–6.9 under an N_2_/CO_2_ atmosphere (80:20). 10 mmol L^-1^ FeCl_2_ was added to all media (regular, NH_4_^+^ deplete, and SO_4_^-^ poor) – Fe(II) concentrations from 200 μmol L^-1^ to 10 mmol L^-1^ have been shown to produce the same growth rates under nutrient rich conditions. The 10 mmol L^-1^ media was filtered after being made to remove any precipitates, which resulted in a final Fe(II) concentration of 2 mmol L^-1^ for the standard media and 4mmol L^-1^ for the NH_4_^+^ deplete media. The low SO_4_^-^ media was left unfiltered with an Fe(II) concentration of 10 mmol L^-1^. In the ammonium free media, NH_4_Cl was replaced with 0.3 g/L KCl and an additional 10 mL of N_2_ gas was injected into the headspace. In the low sulfate media, 0.0025 g/L MgSO_4_ and 0.4 g/L MgCl_2_ were added instead of the usual 0.5 g/L MgSO_4_. Furthermore, approximately 10 kBq of carrier-free ^35^S was added to all of the low sulfate cultures. The cultures for the N-fixation experiments were grown in ammonium free conditions once and then transferred into the final experimental bottles. The culture for the S^35^ experiment was grown up in standard media, spun down and decanted to avoid adding extra sulfate, before the cells were inoculated into the final experimental bottles. All cultures were grown under a constant light intensity of 14 μE m^-2^ s^-1^.

### Analytical Techniques

Spectrophotometric analysis of Fe(II) and Fe(III) concentrations were performed using the ferrozine method; samples were measured directly as well as after being fixed in 1 N HCl – after Viollier et al. (2000). Pigments were measured spectrophotometrically after 24 h extractions of 1 mL of pelleted cells in acetone:methanol (7:2 v/v) (Frigaard et al., 1996). Cells numbers were then obtained using a pigment to cell count conversion factor of 6.3 × 10^-10^ pigment/cell/mL for *C. phaeoferrooxidans* and 5.8 × 10^-10^ pigment/cell/mL for *C. ferrooxidans*. The cells from the ^35^S experiment were collected via filtration along with a liquid sample as a background measurement. The filtered samples were subsequently washed with 5% Trichloroacetic acid (TCA) in order to kill, wash, and dissolve cellular material. TCA precipitates DNA and proteins, leaving only these cellular components on the filter and therefore any counts associated with the filtered samples would indicate ^35^S that had been incorporated into this cellular biomass (Cuhel et al., 1981). Five milliliter of scintillation fluid were added to the ^35^S samples (1 mL of liquid or the filter) and all samples were counted using a scintillation counter.

### Bioinformatics

Genomes of Chlorobi stains used in this paper were retrieved from NCBI under the following accession numbers with the completion percentage of each genome in brackets after the number: NC_008639.1 (99.45%), NZ_AASE00000000.1 (90.71%), NC_007514.1 (97.8%), NC_009337.1 (98.91%), NC_010803.1 (99.98%), NC_002932.3 (97.8%), NC_011027.1 (98.89%), and NC_007512.1(98.91%). Genomes were analyzed using MetaPathways V2.5.1, an open source pipeline for predicting reactions and pathways using default settings^1^ (Konwar et al., 2013, 2015) and using the following databases: MetaCyc-v4-11-07-03 (Caspi et al., 2012), Kyoto Encyclopedia of Genes and Genomes (KEGG-11-06-18) (Kanehisa et al., 2008), SEED-14-01-30^2^, Clusters of Orthologous Groups (COG-13-12-27) (Kaufmann, 2006), Carbohydrate-Active enZYmes (CAZY-14-09-04) (Cantarel et al., 2009), and RefSeq-nr-14-01-18 (Agarwala et al., 2015) databases. Initially, we identified all sequences with a functional assignment affiliated with nitrogen fixation and assimilatory sulfur reduction using the MetaPathways functional annotation table output.

### Phylogenetic Trees for Nitrogen Fixation

Individual NifDKH gene sequences from all organisms outside of the phylum Chlorobi were retrieved from NCBI searches from described strains, concatenated, and then aligned using the package software ClustalX2.1 (Larkin et al., 2007). To rigorously test the evolutionary history of nitrogen fixation multiple tree construction methods [Maximum likelihood (ML) and Maximum parsimony (MP)] were employed. ML and MP trees were constructed in MEGA version 7 (Tamura et al., 2013; Kumar et al., 2016) and all trees bootstrapped 500 times. Bootstrap values are indicated at the nodes.

### Phylogenetic Trees for Assimilatory Sulfate reduction

CysH and CysD/Sat gene sequences from all organisms outside of the phylum Chlorobi were retrieved from NCBI searches from described strains, and then aligned using the package software ClustalX2.1 (Larkin et al., 2007). To rigorously test the evolutionary history of ASR multiple tree construction methods (ML and MP) were employed. ML and MP trees were constructed in MEGA version 7 (Tamura et al., 2013; Kumar et al., 2016) and bootstrapped 500 times. Bootstrap values are indicated at the nodes.

### Phylogenetic Trees for 16S rRNA

16S rRNA sequences were retrieved from strains used in Nif and ASR gene trees from the Silva online database – version 128 (Pruesse et al., 2007; Quast et al., 2012). Only full-length (>1400 bp) sequences were selected, and these were aligned, using the package software ClustalX2.1 (Larkin et al., 2007). To rigorously test the evolutionary history of nitrogen fixation and ASR multiple tree construction methods (ML and MP) were employed. ML and MP trees were constructed in MEGA version 7 (Tamura et al., 2013) and all trees bootstrapped 500 times. Bootstrap values are indicated at the nodes.

## Author Contributions

KT performed all laboratory work, except for biochemical verification of assimilatory sulfate reduction, which was performed by SC; KT, RS, and SC interpreted and analyzed the data with bioinformatic data analysis conducted by AH; KT and SC wrote the paper with input from RS. SH contributed to data interpretation and insights, SC supervised the group.

## Conflict of Interest Statement

The authors declare that the research was conducted in the absence of any commercial or financial relationships that could be construed as a potential conflict of interest.

## Footnotes

1 https://github.com/hallamlab/metapathways2/wiki

2 http://www.theseed.org/
